# Challenges in diagnosis and management of acute hepatic porphyrias: from an uncommon pediatric onset to innovative treatments and perspectives

**DOI:** 10.1186/s13023-022-02314-9

**Published:** 2022-04-07

**Authors:** Matteo Marcacci, Andrea Ricci, Chiara Cuoghi, Stefano Marchini, Antonello Pietrangelo, Paolo Ventura

**Affiliations:** Department of Surgical and Medical Sciences for Children and Adults, Internal Medicine Unit, University of Modena and Reggio Emilia, Via del Pozzo 71, 41124 Modena, Italy

**Keywords:** Acute hepatic porphyria, Porphyrias, Givosiran, SiRNA, Neuropathy, Hereditary diseases, Abdominal pain, Delayed diagnosis, Porphobilinogen, Aminolaevulinic acid

## Abstract

Acute hepatic porphyrias (AHPs) are a family of four rare genetic diseases resulting from a deficiency in one of the enzymes involved in heme biosynthesis. AHP patients can experience potentially life-threatening acute attacks, characterized by severe abdominal pain, along with other signs and symptoms including nausea, mental confusion, hyponatraemia, hypertension, tachycardia and muscle weakness. Some patients also experience chronic manifestations and long-term complications, such as chronic pain syndrome, neuropathy and porphyria-associated kidney disease. Most symptomatic patients have only a few attacks in their lifetime; nevertheless, some experience frequent attacks that result in ongoing symptoms and a significant negative impact on their quality of life (QoL). Initial diagnosis of AHP can be made with a test for urinary porphobilinogen, $\delta$-aminolaevulinic acid and porphyrins using a single random (spot) sample. However, diagnosis is frequently missed or delayed, often for years, because the clinical symptoms of AHP are non-specific and mimic other more common disorders. Delayed diagnosis is of concern as some commonly used medications can trigger or exacerbate acute attacks, and untreated attacks can become severe, potentially leading to permanent neurological damage or fatality. Other attack triggers include hormonal fluctuations in women, stress, alcohol and low-calorie diets, which should be avoided in patients where possible. For the management of attacks, intravenous hemin is approved, whereas new therapeutic approaches are currently being investigated as a baseline therapy for prevention of attacks and improvement of QoL. Among these, a novel siRNA-based agent, givosiran, has shown very promising results in a recently concluded Phase III trial and has been approved for the management of AHPs. Here, we propose a challenging case study-with a very unusual pediatric onset of variegate porphyria-as a starting point to summarize the main clinical aspects (namely, clinical manifestations, diagnostic challenges, and therapeutic management) of AHPs, with a focus on the latest therapeutic innovations.

## Introduction

**Fig. 1:**
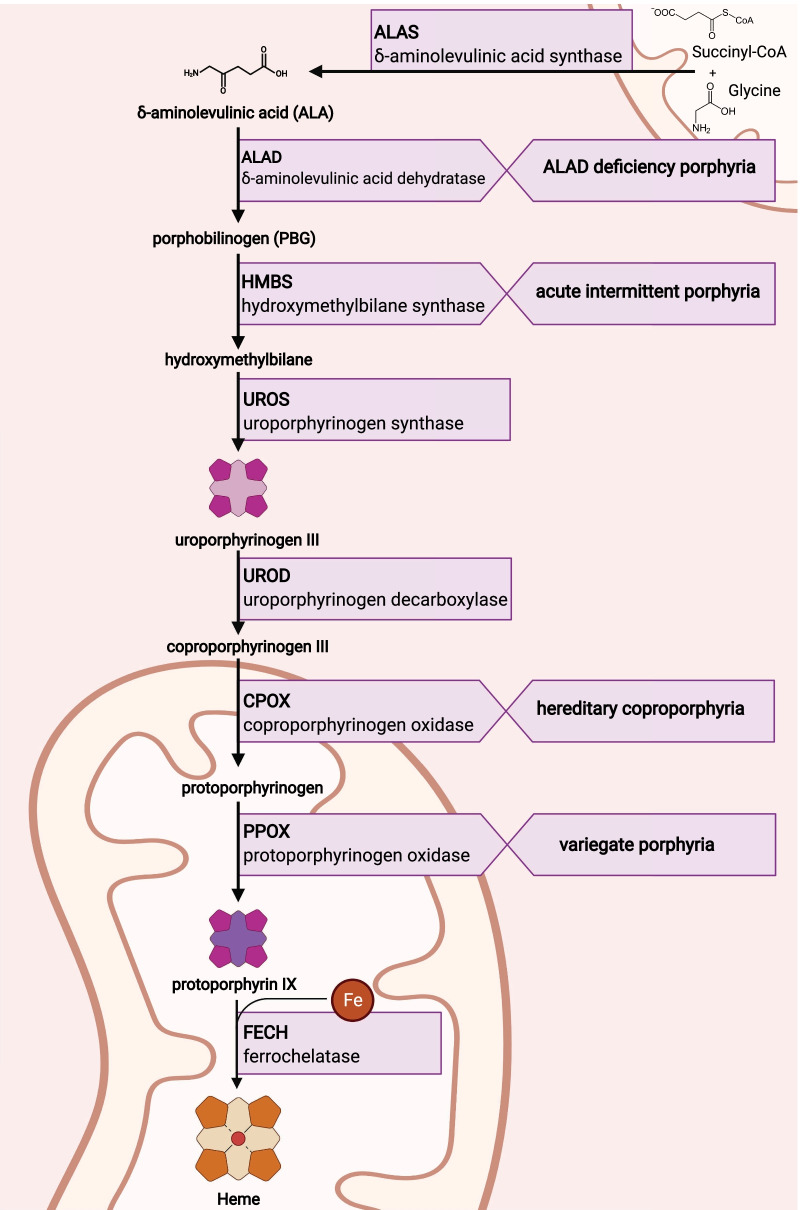
Heme biosynthesis pathway with the corresponding type of acute hepatic porphyria. CoA: coenzyme A; Fe: iron. Created with BioRender.com (last accessed 9 January 2022)

Acute hepatic porphyrias (AHPs) are a family of four rare genetic diseases characterized by potentially life-threatening acute neurovisceral attacks (acute porphyric attacks, APAs) and, for some patients, chronic debilitating manifestations whose burden negatively impacts their quality of life (QoL) [1–3]. The diseases result from a genetic defect leading to a functional impairment in four of the eight enzymes of the pathway of heme biosynthesis (Fig. 1) [4]. The four types of AHP are acute intermittent porphyria (AIP, the most common type), variegate porphyria (VP), hereditary coproporphyria (HCP) and the ultra-rare ALA dehydratase deficiency porphyria (ADP), each resulting from a different enzyme deficiency (Table 1) [5–7].

**Table 1:** Types of acute hepatic porphyrias

Type	Allele and inheritance pattern [8]	Sex of symptomatic patients [11]	Estimated prevalence of symptomatic patients (per million) [12]	Potential symptoms [3, 8]	Important biochemical features [25]
AIP	HMBS^a^ autosomal dominant	Predominantly female	5.9	Acute attacks Chronic	ALA and PBG usually elevated at all times^b^
VP	PPOX autosomal dominant	Predominantly female	3.2	Acute attacks Chronic Cutaneous	ALA and PBG usually elevated only during attacks
HCP	CPOX autosomal dominant	Predominantly female	0.8	Acute attacks Chronic Cutaneous	ALA and PBG usually elevated only during attacks
ADP	ALAD autosomal recessive	All recorded symptomatic patients have been male	Ultra-rare (< 10 documented cases)	Acute attacks Chronic	ALA usually elevated at all times, PBG not usually elevated

^a^ HMBS is also known as PBGD. ^b^ In patients with acute intermittent porphyria, ALA and PBG are usually elevated at all times and typically increase further during attacks. ADP: $\delta$-aminolaevulinic acid dehydratase deficiency porphyria; AIP: acute intermittent porphyria; ALA: $\delta$-aminolaevulinic acid; ALAD: aminolaevulinic acid dehydratase; CPOX: coproporphyrinogen oxidase; HCP: hereditary coproporphyria; HMBS: hydroxymethylbilane synthase; PBG: porphobilinogen; PBGD: porphobilinogen deaminase; PPOX: protoporphyrinogen oxidase; VP, variegate porphyria

Although most heme in the human body is synthesized in the bone marrow for hemoglobin synthesis (75–80%), $\sim$15–20% is synthesised in the liver as a cofactor for numerous hemoproteins (e.g., myoglobin, cytochrome P450s, catalase and peroxidase) [1]. The body’s need for hepatic heme therefore fluctuates depending on exogenous factors, such as medication or alcohol intake, or endogenous factors such as hormone levels [8]. By engendering an increased requirement of heme, these factors can act to up-regulate hepatic $\delta$-aminolaevulinic acid (ALA) synthase (ALA synthase 1, ALAS1), the first and rate-limiting enzyme in the eight-step pathway of heme biosynthesis in the liver (Fig. 1) [7, 8]. In patients with AHP, whose heme biosynthesis is dysfunctional, the upregulation of ALAS1 can lead to increased levels of heme intermediates such as ALA and porphobilinogen (PBG) (Fig. 1) [6]. Accumulation of ALA, and possibly PBG, is believed to be neurotoxic [9] and the primary cause of the disease manifestations of AHP [8, 10]. A variety of triggers can increase heme requirements and activate this process leading to acute attacks, including hormonal fluctuations in women, infections, stress, use of certain medications, alcohol and fasting/low-calorie diets [4, 11].

The prevalence of symptomatic patients with AHP in Europe is estimated at 1:100,000 [12]. Most symptomatic patients have only a few APAs in their lifetime; however, up to 8% will suffer from ongoing attacks (typically defined as at least four APAs per year) [13]. The age of onset of clinical manifestation varies, but is most common between the second and fourth decades of life, whereas onset before puberty is unusual [8, 14], with most reports focusing on pediatric presentations of AIP [15–18]. Although both sexes inherit mutations with equal frequency, women are predominantly affected, likely related to the effect of hormonal changes (e.g., menstrual cycle) [8, 19].

With regard to the most common among AHPs, there is a relatively high incidence of mutations associated with AIP ($\sim$1/1600 Caucasians) but clinically manifest disease occurs in < 10% of the at-risk population, indicating the importance of genetic modifiers and environmental factors [20, 21].

In recent years, the management of acute hepatic porphyrias has been revolutionised by small interfering RNA (siRNA) technology: givosiran (Givlaari$\circledR$), a siRNA-based agent specifically targeting ALAS1 in the liver, has shown excellent results in lowering the annual rate of APAs and improving the overall QoL of patients.

Here, we present a case study as a starting point for discussion of the main clinical aspects (namely, clinical manifestations, diagnostic challenges, and therapeutic management) of AHPs, with a focus on the latest therapeutic innovations.

## Case study

A 14-year-old woman presented to the Emergency Department (ED) due to progressive loss of strength in the limbs with difficulty in standing, mental confusion, crampy severe lower abdominal pain, nausea and vomiting. All symptoms had been progressive in their onset; they started about 30 days after the onset of fever, due to a documented viral infection (mononucleosis). The patient had a history of ED admissions due to recurrent episodes of abdominal pain (with negative radiography, ultrasound and computerized tomography [CT] scans) that were variably responsive to analgesic medications. The patient reported that during past episodes of abdominal pain, she had developed nausea, darkening of urine, fatigue, limb muscle weakness, difficulty with concentration and worsening of constipation, which for her was a chronic problem. The patient mentioned previous episodes of anorexia and weight loss. She also had a history of epilepsy (absence seizures, treated with sodium valproate 600 mg/day and ethosuximide 750 mg/day) and left subcortical parietal cavernoma with venous dysgenesis. No history of sunlight intolerance or skin lesions in sunlight exposed regions was referred by the patient.

In the ED, the body mass index was low (17.2 kg/m$^{2}$; reference range 18.5–25 kg/m$^{2}$), whereas temperature, blood pressure, heart rate and respiratory rate were all within the normal range. Laboratory studies revealed an increase in white blood cells (12.7$\times$10$^{3}$/mm3, reference range 4–10.9 $\times$10$^{3}$/mm$^{3}$ , 75% neutrophils), normocytic anemia (hemoglobin 10.5 g/dL, reference range 13.5–17.5 g/dL, mean corpuscular volume 91 fL, reference range 80–99 fL), decreased serum sodium (110 mEq/L, reference range 135–145 mEq/L) and increased blood urea nitrogen (48 mg/dL, reference range 15–45 mg/dL). Potassium, chloride, bicarbonate, blood sugar and creatinine were all within the normal range. Urinalysis revealed scant white and red blood cells (< 5 per high-power field), trace protein and positive ketones, with urinary glucose not detected. Serum alanine transaminase was 48 units/L (reference range 1–37 units/L) and aspartate transaminase 45 units/L (reference range 1–40 units/L), while serum total protein, bilirubin, lipase and amylase were within the normal range; serum *Helicobacter pylori* antibody and coeliac testing were non-reactive.

The patient looked anxious, lethargic and responded slowly to questions. She was variably oriented to person but not to place and time. She continuously complained about pain and requested pain relief. The abdomen was soft and non-distended with absent bowel sounds. No complaints of increased pain, nor any voluntary guarding could be elicited by deep palpation. A cardiopulmonary examination was normal. A neurological examination excluded any focal deficits, albeit disclosing generalized significant weakness (especially in the lower limbs) with hyporeflexia. She was treated with intravenous (IV) saline and hydromorphone, with only a partial improvement in her symptoms, and was admitted for additional observation.

A contrast-enhanced CT scan of the abdomen and pelvis was normal, showing only retained stool in the colon. The findings were similar to those of three CT scans made during previous ED admissions. Nuclear magnetic resonance imaging of the head did not show any significant abnormalities (except for the known left parietal cavernoma), hypophysis imaging was normal. An electroencephalogram showed slow, irritative diffuse abnormalities.

In light of her gender, young age, recurrent abdominal pain, hyponatremia and seizures, a diagnostic hypothesis of acute hepatic porphyria (AHP) was proposed. Thus, urinary analysis of $\delta$-aminolaevulinic acid (ALA), porphobilinogen (PBG) and porphyrins was undertaken. Urinary PBG was 31.6 $\upmu$mol/mmol creatinine (reference range 0–1.5 $\upmu$mol/mmol creatinine), urinary ALA was 23.3 $\upmu$mol/mmol creatinine (reference range 0–5 $\upmu$mol/mmol creatinine) and total urinary porphyrins were 4414 $\upmu$mol/mmol creatinine (reference range 0–82 $\upmu$mol/mmol creatinine) with coproporphyrins prevalent. As a result, a diagnosis of AHP was formulated. Additionally, fecal porphyrins were assessed (1558 nmol/g, reference range < 200 nmol/g) and a plasma fluorescence scan was performed (positive peak at 625 nm), suggesting that the type of AHP was variegate porphyria (VP).

Sodium valproate and ethosuximide, both drugs known to be triggers for attacks [5], were gradually reduced and stopped, and treatment with IV 10% dextrose and hemin (marketed in Europe as Normosang$\circledR$) was started and continued for 4 days. Hemin was reconstituted with human serum albumin and administered by central venous catheter into a high-flow, large-bore vein, in order to decrease the likelihood of venous side effects (such as thrombosis or thrombophlebitis) [5]. IV caloric supplementation (50% carbohydrate input) was also started. Treatment resulted in a dramatic improvement in the patient’s symptoms (complete remission of pain and neurological symptoms after 3 days), together with progressive normalization of serum sodium, and urinary ALA and PBG levels. Genetic testing showed a heterozygous mutation (c.807 G>A) in the protoporphyrinogen oxidase gene, confirming the diagnosis of VP.

## Clinical manifestations of acute hepatic porphyrias

**Fig. 2:**
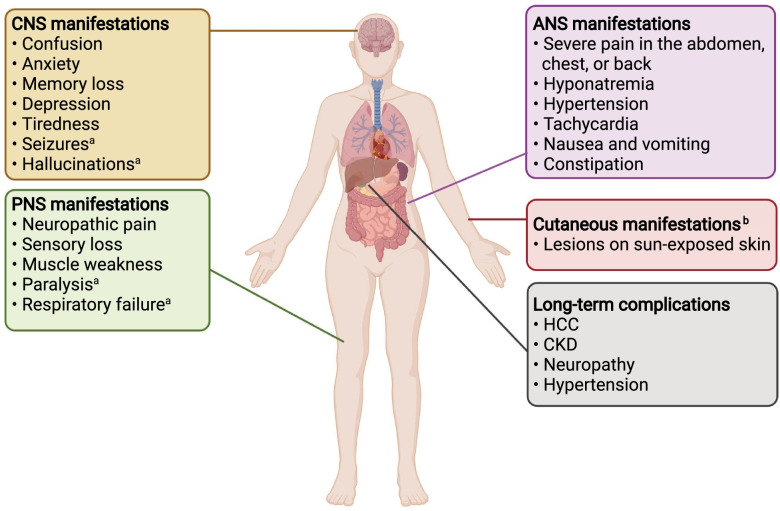
Constellation of clinical characteristics and associated conditions for acute hepatic porphyria. **a** Only occurs in severe attacks. **b** Only occurs in variegate porphyria and hereditary coproporphyria. ANS: autonomic nervous system; CNS: central nervous system; PNS: peripheral nervous system; HCC: hepatocellular carcinoma; CKD: chronic kidney disease. Created with BioRender.com (last accessed 9 January 2022)

The most dramatic manifestations of AHPs are acute neurovisceral attacks, which often require hospitalization and, in the severest cases, a critical care setting [22]. As highlighted in the case study, the most common symptom in AHPs is severe, diffuse abdominal pain; other signs and symptoms can include nausea, weakness, tachycardia, hyponatremia, mental status changes, hypertension and changes in urine colour (Fig. 2) [3, 23]. If the attack is particularly severe, treatment is not initiated promptly or exposure to triggers is prolonged, patients can also experience seizures, delirium and paralysis, posterior reversible encephalopathy syndrome (PRES) along with permanent neurological damage or fatality [1, 5, 24].

Several hypotheses have been proposed to explain the pathogenesis of APAs; some mechanisms of neuronal damage are likely related to the neurotoxic effects of the accumulation of non-porphyrin precursors (PBG, but especially ALA) on the central, peripheral and autonomic nervous systems [2, 9, 25]. Otherwise, additional mechanisms of damage have been hypothesized, such as those supposedly related to relative dysfunctions of the secondary routes of heme utilization (e.g. for the functioning of cytochromes, nitric oxide synthases, or enzymes involved in tryptophan metabolism) [9].

Research has also highlighted that some patients experience chronic symptoms (such as pain, fatigue and nausea) that affect daily functioning and QoL [3, 23, 26]. Additionally, patients with AHP can be at risk of experiencing numerous, multi-system, long-term complications and comorbidities as a result of the disease [22]. Long-term complications related to AHP and its treatment can include liver disease (e.g., hepatocellular carcinoma, fibrosis and cirrhosis), chronic kidney disease, peripheral neuropathy, chronic pain and systemic arterial hypertension [23, 27–32]. Comorbidities related to AHP can include anxiety, depression, elevated lipase/amylase levels, pancreatitis, hypertension, tachycardia and cardiac arrhythmias [3, 22, 31, 33]. In addition, the overproduction of porphyrins in HCP and especially in VP may cause chronic, blistering, photosensitive skin rashes [7, 11].

## Impact on QoL and financial burden

For patients who experience ongoing attacks, AHP can have a substantial negative impact on QoL [26]. Patients have reported diminished QoL compared with population norms, with impacts on multiple aspects of their lives including pain and discomfort, anxiety and depression, the ability to perform usual activities and sleep disorders [3]. Patients have also reported a considerable impact on their social lives, including isolation, missing important occasions and limiting travel [26, 34, 35]. In addition, patients can experience substantial economic burden: many are not fully employed (and receiving disability payments), whereas those who are employed often miss many days of work due to AHP [35]. Patients with symptomatic AHP may also have increased levels of healthcare utilization, notably ED visits and hospital stays [3, 36, 37].

## Diagnosis

As highlighted in the prior case study, the diagnosis of AHP is challenging for several reasons [38]. As a group of rare diseases, AHP is often not considered as part of the differential diagnosis when assessing for acute abdominal pain (and other common symptoms) [25]. Patients experiencing acute attacks often present to EDs where rare diseases may go unnoticed due to time constraints and the priority placed on stabilizing patients [39]. Furthermore, AHP has a variable presentation with many of its symptoms mimicking other, more prevalent conditions, thus making it a difficult disease to identify, even when symptoms are severe [4, 27]. This may be especially true for specialists who focus on a specific organ system, as often, for AHP to be suspected, the totality of a patient’s clinical presentation needs to be considered. This symptom variability and lack of specificity can lead to missed diagnosis or misdiagnosis, often for years, with one study finding a mean delay from onset of symptoms to diagnosis of $\sim$15 years [23].

It should be emphasized that our Case Study features a remarkably early age of onset for variegate porphyria: pediatric presentation of heterozygous AHPs is unusual [14], and most commonly described for AIPs [15, 16]. The exceedingly rare homozygous variants of AHPs, on the contrary, usually present in childhood and tend to manifest with chronic neuropathy, growth retardation, and/or other symptoms of variable severity [14]. It is worth noting that we could not retrieve any explicit report of a case of heterozygous variegate porphyria with pediatric onset from the scientific literature.

**Fig. 3:**
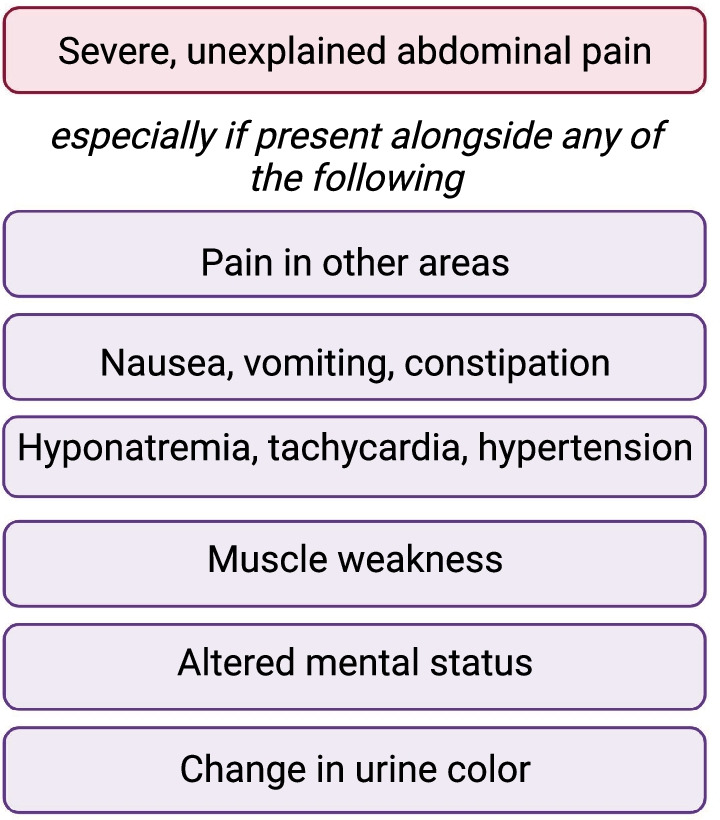
Key symptoms and signs indicating an acute hepatic porphyria attack. Created with BioRender.com (last accessed 9 January 2022)

A timely diagnosis of AHP is crucial as untreated acute attacks can progress, become more severe and potentially lead to permanent neurological damage, or even be life-threatening [25, 40]. Also of concern is that many commonly used drugs can increase hepatic heme requirements, and may trigger acute attacks or exacerbate symptoms (a concern highlighted in the case study) [8]. Undiagnosed patients may therefore inadvertently be prescribed medications that induce or worsen attacks [27]. In addition, undiagnosed patients can be misdiagnosed and given unnecessary medical treatments, and even surgery [5, 23]. AHP should be considered in patients with severe unexplained abdominal pain (which occurs in >90% of acute attacks [3]), particularly if present alongside any of the following signs and symptoms: pain in other parts of the body, nausea, constipation, mental confusion, change in urine color, muscle weakness, hyponatremia, tachycardia and hypertension (Fig. 3).

Biochemical diagnosis of the disease can be undertaken using a random (spot) urine test for PBG, ALA and porphyrins. The optimal time to take a urine sample is during or shortly after an attack, when ALA or PBG levels will be most elevated [25]. PBG levels are particularly useful as they are elevated during AHP attacks (often many orders of magnitude above normal) but not in any other medical condition, allowing the diagnosis or exclusion of AHP [6, 25]. The exception to this is ADP, where ALA but not PBG levels are typically elevated (this can also occur with lead intoxication), although ADP is ultra-rare with < 10 documented cases worldwide [6, 27]. Measurement of urine porphyrins may be important to ensure that a diagnosis of VP or HCP is not missed, as urine PBG excretion can return to normal within a few days of clinical presentation in these two porphyrias (Table 1) [41]. However, a test of urine porphyrins by itself cannot diagnose AHP [6]. Genetic testing of the AHP genes should be undertaken to determine AHP type [42]. All AHP attacks are treated in the same manner, so there is no need to wait for results of genetic testing before initiating the treatment of an attack [6].

## Current treatment approaches

All potentially precipitating factors such as porphyrinogenic medications (a database of safe and unsafe medications can be found here: www.drugs-porphyria.org), reduced calorie intake, smoking and alcohol should be eliminated during an acute attack, and minimized to prevent future attacks [4]. As suggested by the case study, infections may also deteriorate the patient’s clinical condition and should be treated properly [22]. For the management of attacks, IV hemin is approved, due its effectiveness in replacing the heme pool and down-regulating heme biosynthesis [43]. It is usually infused daily (3–4 mg/kg) into a large peripheral vein or venous access port for 3–4 consecutive days, but a repetitive course may be required if AHP symptoms are ongoing [22, 44]. Reconstitution with human serum albumin may reduce the risk of side effects [5]. The treatment should be started immediately during a severe or moderate acute attack after the demonstration of typical symptoms of acute porphyria and an elevation in urine PBG [22]. Side effects of hemin utilisation include thrombophlebitis (for which infusion through a large-bore venous catheter is usually needed), headache, dizziness (ethanol is one of the main excipients) and (hepatic) iron accumulation due to repeated infusions. As presented in the case study, IV glucose may also down-regulate the heme biosynthesis pathway and may be effective, especially in patients who are malnourished or in whom dietary restrictions have contributed to an attack [6]. Being more readily available in most emergency settings, IV glucose may be started early in the attempt to stop the progression of mild attacks, even though great care should be taken not to worsen hyponatremia with the sodium-free content of glucose infusions. Hemin is more effective than glucose in the treatment of acute attacks and should be promptly requested from a (previously designed) pharmacy of reference when a known porphyric patient presents to the ED. In the most severe cases, glucose and hemin infusions may be used together. Finally, it is paramount to strictly monitor the clinical course of the attack, since patients with deteriorating conditions should be promptly moved to a critical care setting.

As pain is a cardinal symptom of AHP, patients often require the use of analgesic regimes, with many patients relying on opioids to manage acute and chronic pain [5, 22]. However, their use, particularly in a chronic setting, needs to be weighed against the risks of addiction, somnolence and apnea [4, 22]. Patients have also expressed concerns around being labelled as malingerers and drug-seekers due to their substantial need for pain relief [26, 35]. Other symptomatic therapy for hypertension, tachycardia, nausea and vomiting is commonly required. [22]

A particular challenge is the treatment of patients who experience ongoing attacks [5]. Current treatment options focus on acute attack management and the resolution of symptoms. Prophylactic approaches remain limited, are used variably and are highly dependent on clinical experience [4]. Off-label prophylactic hemin infusions have been used successfully in some patients. This often requires indwelling central venous catheters, may induce dependence on endogenous heme, and may lead to tachyphylaxis and side effects such iron overload, thrombosis or phlebitis [3, 5, 22, 45]. Chemically induced menopause with gonadotropin-releasing hormone agonists has been used successfully in some young women experiencing acute attacks related to their menstrual cycles [46, 47]. One treatment used in severely affected patients is liver transplantation. Although this is potentially curative, it is rarely used due to the highly invasive nature of the surgery, the need for lifelong immunosuppression and a shortage of donors [48]. Thus, there remains a high unmet medical need for effective treatments for these patients.

## Emerging therapies

Given the limitations of the current therapeutic landscape, novel approaches for the development of efficacious and safe AHP therapies are needed. An investigational RNA interference therapeutic, givosiran, that specifically targets ALAS1 in hepatocytes has been recently approved for the treatment of AHP in adults (in the US and Brazil) or in patients who are older than 12 years (in Europe) [49–51]. Givosiran is subcutaneously administered and has been developed to reduce the overproduction of potentially neurotoxic heme intermediates in the liver [39, 52]. Results from a Phase III clinical study (ENVISION) in AHP patients with recurrent attacks showed, compared to placebo, a lower attack rate, less debilitating symptoms and an improved QoL between attacks, significantly decreased levels of ALA and PBG, and an acceptable safety profile [53, 54]. Givosiran has been approved for the treatment of patients with AHP, regardless of their annualized attack rate, albeit its efficacy has been tested mainly in patients with a clinically active disease and more frequent and severe APAs: in this population and to the authors’ opinion, givosiran has represented a real breakthrough in the prevention of potentially life-threatening attacks. Among the most frequently reported adverse effects, special attention should be paid to hyperhomocysteinemia (however responsive to vitamin supplementation therapy) [55–57]. Also reported were injection-site reactions and elevations in liver transaminases and pancreatic enzymes. Recently, a decline in renal function was detected in a minority of patients under siRNA-based therapy, which was worse than expected given the natural course of porphyria-associated kidney disease [58]. For these reasons, we suggest that every patient should have a blood chemistry check-up inclusive of liver transaminases, pancreatic enzimes, kidney function, and homocysteine, before starting therapy with givosiran and periodically (i.e. every few weeks) thereafter. In particular, increases in homocysteine should be promptly treated as already described [57].

In another approach to find a treatment for AHP, gene therapy with a viral vector delivering a normal hydroxymethylbilane synthase (HMBS) gene to hepatocytes was assessed in a Phase I clinical trial. Despite an acceptable safety profile, this investigational agent did not show efficacy in preventing frequent attacks [59]. Further research is underway to optimize the viral vector [60].

Finally, in a pre-clinical study, intravenous human HMBS messenger RNA encapsulated in lipid nanoparticles was used to transduce hepatocytes. In AIP mice, this approach reduced urinary heme intermediates when acute attacks were induced, suggesting that it may be a potential therapy for AIP [61]. A clinical trial is necessary to demonstrate the safety, feasibility and efficacy of this therapy in humans.

## Conclusion

AHP is a family of rare, serious diseases resulting from a genetic defect in the heme biosynthesis pathway enzymes in the liver. Patients can experience potentially life-threatening acute attacks, chronic manifestations and long-term complications. Accumulation of heme precursors ALA, and possibly PBG, are believed to be neurotoxic and the primary cause of disease manifestations.

Diagnosis is challenging due to a non-specific, variable presentation with many of the symptoms mimicking other, more prevalent conditions. However, a relatively straightforward biochemical test for urinary PBG, ALA and porphyrins using a single random (spot) sample can exclude or provide an initial diagnosis of AHP. Most symptomatic patients have only a few attacks in their lifetime; nonetheless, some experience frequent attacks with up to 8% having four or more attacks per year. As a result, these patients experience a strong negative effect on their QoL and have high unmet needs for new treatment options. Results of the Phase III trial suggest that givosiran may be an effective treatment to reduce attacks in these patients.

## Data Availability

Not applicable

## Abbreviations

ALA: $\delta$-aminolaevulinic acid
ALAS1: ALA synthase 1
AHP: acute hepatic porphyria
APA: acute porphyric attacks
CT: computerised tomography
ED: Emergency Department
IV: intravenous
PBG: porphobilinogen
QoL: quality of life
RNA: ribonucleic acid
siRNA: small interfering RNA
VP: variegate porphyria
